# Prevalence of Self-Reported Muscle Pain Among Statin Users From National Guard Hospital, Riyadh

**DOI:** 10.7759/cureus.23463

**Published:** 2022-03-24

**Authors:** Ihab Suliman, Abdulaziz Batarfi, Hassan Almohammadi, Hisham Aljeraisi, Hassan Alnaserallah, Ali Alghamdi

**Affiliations:** 1 Cardiology, Cardiac Center, King Abdulaziz Medical City, Riyadh, SAU; 2 College of Medicine, King Saud Bin Abdulaziz University for Health Sciences, Riyadh, SAU

**Keywords:** rhabdomyolysis, ischemic heart disease, cardiac intervention, statin, muscle pain

## Abstract

Background

Statin, a hydroxymethylglutaryl-coenzyme A reductase inhibitor, is one of the commonly used lipid-lowering drugs that is used for lowering lipid levels in the body. Muscle pain is a commonly reported adverse effect of statins, yet little is known about the prevalence of muscle pain and statin use in the general population.

Methods

The cross-sectional study was conducted in National Guard Hospital, Riyadh, Saudi Arabia. All study subjects were adult statin users aged 18 years old or above. A total of 313 patients were included in the study. The study was conducted based on a questionnaire distributed among patients according to inclusion and exclusion criteria.

Results

Among 313 statin users, patients underwent cardiac catheterization (39, 12.5%), stress test (62; 19.8%), percutaneous coronary intervention (three; 1.0%), and coronary artery bypass graft (six, 1.9%), while 203 patients didn’t have any intervention (64.9%). Most of the study subjects were on atorvastatin (139; 44.4%).

The prevalence of muscle pain was 73.5%; 95% CI = (68.4% - 78.1%). The most common sites of pain were lower limb pain (160; 51.1%), upper limb pain (145; 46.3%), and trunk pain (96; 30.7%). The common types of pain were joint pain (52; 16.6%), muscle weakness (51; 16.3%), muscle aches (43; 13.7%), and muscle cramps (41; 13.1%); and patients who reported that they stopped statin at some point because of muscle pain were 92 (29.4%).

Conclusion

Statins are important for managing and preventing ischemic heart diseases. Our study found that muscle pain is highly associated with statin use with a prevalence of 73.5%, which causes many patients to tend to stop taking their medication. Therefore, preventing the side effects by adjusting the proper dose or switching to another type of statin for high-risk patients will help them to continue using the drug. Also, it is important to rule out secondary causes of myopathy such as physical activity, fracture, thyroid dysfunction, or infection.

## Introduction

Ischemic heart disease (IHD) is a common and important disease that must be controlled. Ischemia is defined as a "restriction of blood flow and thus oxygen in a part of the body due to narrowing of the blood vessel." [1]. Coronary artery disease (CAD) narrowing can be caused by a blood clot, constriction of the blood vessel, or as in most cases, caused by a buildup of plaque. When there is a complete obstruction of the blood flow to the cardiac myocytes, the heart muscle cells die, which can result in a heart attack or myocardial infarction (MI) [1]. Most people with slight narrowing of the vessels do not experience symptoms of ischemic heart disease. However, as narrowing progresses, especially if it is untreated, symptoms may occur. They are most likely to occur during exercise or secondary to emotional stress when there is an increase in body demand for oxygen [1]. 

Nowadays statin is considered standard practice for management after acute coronary syndrome, regardless of lipid levels [2]. Statin, a hydroxymethylglutaryl-coenzyme A (HMG-COA) reductase inhibitor, is one of the antihyperlipidemic drugs used for lowering lipid levels in the body [2]. These drugs appear to inhibit the synthesis or promote the breakdown of cholesterol. They are used to treat hyperlipidemia when non-pharmacological treatment failed to reduce cholesterol levels, and they are used in the prevention and treatment of cardiovascular disease [3, 4]. However, they can be associated with many side effects, such as myalgia, myopathy, rhabdomyolysis, and diabetes mellitus. Therefore, many patients frequently stop statin therapy without medical consultation secondary to the side effects, and therefore they are at higher risk for cardiovascular events [5-7]. Myopathic effect with muscles weakness can occur with a normal or minor increase in creatine kinase (CK). Activity in plasma is observed in some patients receiving statins, frequently associated with heavy physical activity. Rarely, the patient may have a marked elevation in CK activity, often accompanied by generalized discomfort or weakness in skeletal muscles. If the drug is not discontinued, myoglobinuria can occur, leading to serious injury [8-10]. Common factors that will lead to the development of muscle pain include age more than 75 years old, gender, alcohol abuse, renal and hepatic dysfunction, and the use of drugs inhibiting the metabolism of statins such as erythromycin [3]. Moreover, genetic variation in anion-transporting polypeptides 1B1 (OATP1B1) is associated with severe myopathy and rhabdomyolysis induced by statins [11-14].

Globally, there are few studies exploring this problem [15, 16]. For example, a study reported the prevalence of muscle pain to be 27.9% in patients taking statins; as a result, clinicians had to adjust the dose or use alternative statins to minimize the side effects [15]. Another cross-sectional study at the Punjab Institute of Cardiology, Pakistan, reported that 51% of patients had myalgia, that it was more common in patients aged 40-50, and that the female to male ratio was 57:47, showing higher susceptibility in females compared to males [16]. Also, it appears that the prevalence of myalgia and an increase in creatine phosphokinase (CK) levels were dose-related. In other words, an increase in the doses would lead to an increase in muscle-related problems. Also, it has been shown that myalgia is associated with the duration of statin use [16].

There are many details required to be covered in the future, including identifying the dosage of statins that triggers muscles pain. Moreover, there are many subtypes of statin, so we must discover which subtype causes more serious muscle pain than others, and also whether muscle pain is associated with other comorbidities unrelated to statin use or not. These points may be used for the initiation of new studies in the future. 

Because there is a lack of research in Saudi Arabia about this problem, we are conducting this research to determine the prevalence of self-reported muscle pain among statin users attending National Guard Hospital, Riyadh. Moreover, we will assess the compliance of statin due to self-reported muscle pain, and we will determine which musculoskeletal regions are affected by statin muscle pain.

## Materials and methods

Study design and area and settings

This is a cross-sectional study to measure the prevalence of self-reported side effects of statins amongst the selected population, conducted in National Guard Hospital, Riyadh, Saudi Arabia.

Identification of study participants

The study about the prevalence of self-reported muscle pain included 313 patients who were using statins. It included patients on statins who were above 18 years of age with or without cardiac intervention such as cardiac catheterization, coronary artery bypass grafting (CABG), or percutaneous coronary intervention (PCI). We used the non-probability convenient sampling method as we had chosen our study population according to certain characteristics.

Data collection process

The data was collected using a self-administered questionnaire to patients who are using statins and attending the National Guard Hospital. The questionnaire contained different types of questions to determine the prevalence of a specific side effect of a drug, which region of the body is more susceptible, which drug is being used, and how many patients have stopped using the drug due to this side effect. The questionnaire contained 14 questions, and they were translated into Arabic by group members. The questionnaire was reviewed by experts in the cardiac clinic to test for its content validity and reliability. Also, we used in our questionnaire some questions from Statin Experience Approved Questionnaire [17]. After the translation, the questionnaire was given to 10 people who were not part of the study to check for difficulties in understanding questions and the average time it takes to finish the questionnaire. The awareness was measured quantitatively by the number of questions answered correctly. Finally, the grouping variables chosen were gender, age, and educational level.

Data analysis

Data was entered and analyzed using SPSS Statistics v. 24.0 (IBM Corp., Armonk, NY). The descriptive statistics were presented as frequency and percentage for the categorical variables e.g. (Gender, education level) and the mean ± SD for numerical variables. Chi-square was used for the association between body pain and patients’ characteristics. A test was significant if p-value < 0.05.

## Results

The questionnaire responders were N=313; 163 (52.1%) were males and 150 (47.9%) were females (Table 1). The overall mean age was 55.1±11.5. Among 313 statin users, 39 (12.5%) patients underwent cardiac catheterization, 62 (19.8%) stress echo, three (1.9%) percutaneous coronary intervention, and six (1.9%) coronary artery bypass graft (Table 2), while patients who didn’t have any interventions were 203 (64.9%). Atorvastatin, with 139 (44.4%) users, was the most used drug, followed by rosuvastatin (44; 14.1%), and simvastatin (22; 7%). Additionally, 101 (32.3%) patients used other statins and seven (2.2%) didn’t know the name of the drug (Table 3).

**Table 1:** Presence of muscle pain with respect to demographic characteristics

Demographic Characteristics	Body pain				
	No		Yes		
Gender	N	%	N	%	P-value
Male	48	29.4	115	70.6	0.221
Female	35	23.3	115	76.7	
Age group					
< = 40	10	25.6	29	74.4	<0.001
41 - 50	26	46.4	30	53.6	
51 - 60	19	16.1	99	83.9	
61+	28	28	72	72	
Educational level					
Non-educated	7	15.9	37	84.1	0.007
Elementary school	15	38.5	24	61.5	
Middle school	2	10.5	17	89.5	
High school	20	44.4	25	55.6	
Higher diploma	4	23.5	13	76.5	
Bachelors	24	20.9	91	79.1	
Master or doctorate degree	11	32.4	23	67.6	

**Table 2:** Demographic characteristic of study subjects

	Demographic characteristics	N	%
Age group	< = 40	39	12.5
	41 - 50	56	17.9
	51 - 60	118	37.7
	61+	100	31.9
Educational level	Non-educated	44	14.1
	Elementary school	39	12.5
	Middle school	19	6.1
	High school	45	14.4
	Higher diploma	17	5.4
	Bachelors	115	36.7
	Master or doctorate degree	34	10.9
Diagnostic test	Stress test	62	19.8
	Percutaneous coronary intervention (PCI)	3	1.0
	Coronary artery bypass grafting (CABG)	6	1.9
	Cardiac catheterization	39	12.5
	None	203	64.9
Drug	Atorvastatin (Lipitor)	139	44.4
	Rosuvastatin (Crestor)	44	14.1
	Simvastatin (Zocor)	22	7
	Others	101	32.3
	Don’t know	7	2.2

**Table 3:** Severity and type of pain with respect to the type of statin used

		Atorvastatin (Lipitor)		Rosuvastatin (Crestor)		Simvastatin (Zocor)		Others		Don’t know	
Type of pain	Severity	N	%	N	%	N	%	N	%	N	%
Severity of muscle aches (muscles feeling sore, strained, or stiff).	None	41	29.5	9	20.5	7	31.8	32	31.7	1	14.3
	Mild	24	17.3	9	20.5	10	45.5	20	19.8	3	42.9
	Moderate	53	38.1	24	54.5	3	13.6	33	32.7	1	14.3
	Severe	21	15.1	2	4.5	2	9.1	16	15.8	2	28.6
Severity of muscle cramps.	None	55	39.6	13	29.5	9	40.9	48	47.5	0	0
	Mild	32	23	16	36.4	9	40.9	18	17.8	3	42.9
	Moderate	30	21.6	13	29.5	2	9.1	20	19.8	4	57.1
	Severe	22	15.8	2	4.5	2	9.1	15	14.9	0	0
Severity of muscle weakness (feeling of heaviness or tiredness in the muscle)	None	43	30.9	13	29.5	6	27.3	38	37.6	0	0
	Mild	35	25.2	6	13.6	10	45.5	16	15.8	0	0
	Moderate	39	28.1	23	52.3	4	18.2	22	21.8	7	100
	Severe	22	15.8	2	4.5	2	9.1	25	24.8	0	0
Severity of joint pain	None	34	24.5	12	27.3	4	18.2	31	30.7	0	0
	Mild	31	22.3	8	18.2	6	27.3	22	21.8	0	0
	Moderate	50	36	21	47.7	10	45.5	29	28.7	3	42.9
	Severe	24	17.3	3	6.8	2	9.1	19	18.8	4	57.1

**Figure 1:**
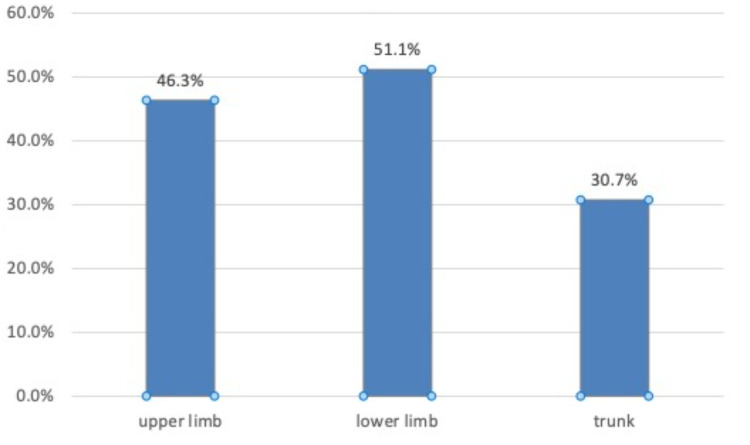
The distribution of the site of pain in statin users

The prevalence of muscle pain was 73.5%; 95% CI = (68.4% - 78.1%). The most common sites of pain were lower limb pain (160; 51.1%), upper limb pain (145; 46.3%), and trunk pain (96; 30.7%) (Figure 1). Common types of pain were joint pain (52; 16.6%), muscle weakness (51; 16.3%), muscle aches (43; 13.7%), and muscle cramps (41; 13.1%). The number of patients who reported that they stopped statin at some point because of muscle pain was 92 (29.4%) while 221 (70.6%) patients reported that they will most likely continue using statin despite having muscle pain.

## Discussion

Statin-induced myopathy is a commonly reported adverse effect. In this study, we found that the prevalence of muscle pain was 73.5%; 95%CI = (68.4% - 78.1%). Around 76.7% of female statin users and 70.6% of male statin users reported pain. This means that statin-induced myopathy is slightly more prevalent in females. In comparison to our study, there is another cross-sectional study done in 2018 which studied the prevalence of statin-induced myopathy at Punjab Institute of Cardiology, Lahore, Pakistan. The results showed that 51% of patients were myalgic [16]. In addition to that, muscle pain was more common in patients aged between 40-50, with females 57% and males 47%, so it is concluded that females were more prone to have muscle pain compared to males [16]. Furthermore, a prospective cohort study conducted at the National Center for Diabetes, Endocrinology, and Genetics (NCDEG) in Jordan showed that the overall incidence of myopathy statin users was 27.8%. Also, the incidence of statin-induced myopathy was highest with simvastatin 40 mg (50%) and lowest with fluvastatin XL 80 mg (8%) and rosuvastatin 10 mg (10.8%) [15]. On the other hand, in our study, we found that muscle pain was prevalent in 79.5% of rosuvastatin users, 70.5% of atorvastatin users, and 68.2% of simvastatin users.

Nowadays statin is considered standard practice for management after acute coronary syndrome regardless of lipid level [2]. Statin is one of the antihyperlipidemic drugs used to lower lipid levels in the body [2]. It acts as an inhibitor for HMG-COA reductase. Along with this drug’s beneficial outcomes, it also has risks, and most found is myopathy [18]. Myopathy ranges from myalgia (muscle symptoms with normal creatinine kinase levels) to myositis (muscle symptoms with elevated creatinine kinase levels). In rare situations, it can worsen to life-threatening rhabdomyolysis (muscle symptoms with markedly elevated creatinine kinase levels and myoglobinuria) [6].

This present study shows that patients on statins have muscle pain in various body parts, manifesting most commonly in the lower limbs, upper limbs, trunk, and back. The symptoms vary in severity from mild to moderate to severe muscle pain. Other symptoms reported are muscle cramps, muscle weakness, and joint pain.

Among statin users, we included patients that had one or more of these interventions, namely cardiac catheterization, stress test, percutaneous coronary intervention, coronary artery bypass graft, or those who didn’t have any intervention. A minority of patients reported that they stopped statin at some point because of muscle pain; this may affect their adherence to the medications, consequently leading to abnormalities in their cholesterol levels.

## Conclusions

Statin is important for managing and preventing ischemic heart diseases. Muscle pain is highly associated with statin use with a prevalence of 73.5%. As a result, patients tend to stop taking their prescribed medications. Therefore, preventing the side effects by adjusting the proper dose or switching to another type of statin for patients at high risk will help them to continue using the drug. Also, it is important to rule out secondary causes of myopathy such as physical activity, fracture, thyroid dysfunction, or infection.
